# International Hahnemann Association

**Published:** 1899-04

**Authors:** B. Fincke

**Affiliations:** Chairman of Homœopathic Philosophy; Brooklyn, N. Y.


### INTERNATIONAL HAHNEMANN ASSOCIATION.

Brooklyn, N. Y., March 25th, 1899.

*To the Members of The International Hahnemannian Association:*

Though the time before the next meeting of the I. H. A. is rather a short one, yet it might be deemed appropriate to direct your attention to a subject in homœopathic philosophy, which you are requested to place in a clearer light by your contribution to this bureau.

The life of a scientific association does not consist so much in the multitude of members and in their personal participation in the meetings as desirable as this naturally must be, as in the spiritual endeavor animating the whole body in considering the fundamental principles again and again by the light of critical analysis, which is apt to infuse fresh vigor into the organization.

Long since the old school has thrown out of its sacred precincts the life-force of Hahnemann, as being an idea without object and reason, just as it also ignores the high potencies of Hahnemann and his disciples as mere figments of the brain.

In later times the protoplasmic cell of man has by these searchers of truth been raised to the dignity of the rejected life-force. They transferred its attributes upon that simple cell, even endowing it with mind, and in this manner in conjunction with the subsequent millions of millions of companions in the signification of vital units they made it the creator of the human organism.

Now from the standpoint of a homœopathician, it is inconceivable how a high potency such as a hundred thousand or million, or even a Hahnemannian thirtieth, can act upon the whole body in health, as a proving, and in disease, as a cure, if the conception of a life-force, as Hahnemann delineated it, is excluded from his philosophy.

You are therefore respectfully invited as a member of an Association which stands for the minimum dose and consequently for high potencies to assist in defining and defending the Hahnemannian position against this monocellular theory (a counterpart of the atomic theory), which likewise conceals the old enemy of Homœopathy—Materialism.

But any other subject of your choice will also be welcome.

Yours fraternally,

B. Fincke, M. D.,

*Chairman of Homœopathic Philosophy.*

